# Crosstalk between Hedgehog pathway and energy pathways in human adipose-derived stem cells: A deep sequencing analysis of polysome-associated RNA

**DOI:** 10.1038/s41598-018-26533-y

**Published:** 2018-05-30

**Authors:** Patrícia Shigunov, Lucas Titton Balvedi, Marlon Dias Mariano Santos, Roberto H. Herai, Alessandra Melo de Aguiar, Bruno Dallagiovanna

**Affiliations:** 1 Laboratory of Basic Biology of Stem Cells (LABCET), Instituto Carlos Chagas - FIOCRUZ-PR, Curitiba, Paraná, 81830-010 Brazil; 2 Computational Mass Spectrometry & Proteomics Group – Instituto Carlos Chagas – FIOCRUZ – PR, Curitiba, Paraná, 81830-010 Brazil; 3 Experimental Multiuser Laboratory (LEM), Cellular Therapy Division, Graduate Program in Health Sciences, School of Medicine, Pontifícia Universidade Católica do Paraná (PUCPR), Curitiba, Paraná, 80215-901 Brazil

## Abstract

Adult stem cells are considered promising candidates for cellular therapies due to their capacity to differentiate and self-renew. Differentiation leads to changes in the metabolism, structure, and gene expression patterns of cells. Hedgehog is one of the pathways that is involved in the enhancement of osteogenesis and chondrogenesis in adult stem cells, but its mechanisms are poorly understood. In this study, we treated adipose tissue-derived stem cells (ADSC) with two well-characterized drugs, purmorphamine (Hedgehog pathway activator) and cyclopamine (Hedgehog pathway inhibitor), and identified mRNAs associated with polysomes in each treatment group to determine the post transcriptional genetic networks governed by the Hedgehog pathway. Activation of the Hedgehog pathway by purmorphamine results in significant upregulation of mRNAs associated with cellular communication and signal transduction. Furthermore, our experiments show that cyclopamine acts late downregulating *GLI1* expression in ADSCs but promotes the upregulation of mRNAs associated with energy pathways and metabolism at early times. Through *in silico* analysis, we identified some miRNAs, such as miR-355, that could regulate these mRNAs association with polysomes and thereby modulate the Hedgehog pathway. Our results suggest that activation of the Hedgehog pathway by purmorphamine also results in a negative regulation of mRNAs in the protein translation machinery.

## Introduction

Cell signaling is a complex system of communication that governs basic functions of cells and coordinates cell actions^1^. The ability of cells to perceive and correctly respond to their microenvironment is the basis of development, tissue repair, immunity, and tissue homeostasis. Studies regarding signaling pathways have traditionally focused on delineating immediate upstream and downstream molecular interactions. These interactions are then organized into linear cascades that relay and regulate information from cell surface receptors to cellular effectors, such as metabolic enzymes, channel proteins, or transcription factors^2^.

The activation of transcriptional factors is a key step in the control of gene expression. Some pathways, show a well -defined sequence of events such as a signaling molecule that binds to the receptor, triggering the intracellular transduction will result in the activation of a transcriptional factor responsible for expressing specific genes. Additionally, transcriptional regulation is the first of the several regulatory step before mRNA is translated into a protein.

The Hedgehog (Hh) pathway has a well-studied cascade of events where the extracellular activating molecules (Sonic, Indian, and Desert Hh)^3^, the receptors (Patched 1 – PTCH1 and Patched 2 – PTCH2), intracellular transduction molecules (Smoothened - SMO, Suppressor of fused homolog - SUFU, and Glycogen synthase kinase 3 beta - GSK3β)^4^, transcription factors (GLI family zinc finger 1, 2 and 3 - Gli1, Gli2, and Gli3)^5,6^ and induced genes (cyclin D, cyclin E, Gli1, and MYC proto-oncogene) are known. However, the post-transcriptional steps involved in the regulation of this pathway are poorly understood.

Since its original discovery in *Drosophila*, Hh protein family members have been identified in all vertebrates. In mouse and human adult stem cells, this pathway is responsible for cell differentiation, as it shows proosteogenic and antiadipogenic properties in several types of mesenchymal stem cells^7–11^. Recent studies have identified Hh signaling in the morphological transition to pre-osteoblasts, exploring the global kinase profile associated with this event^11^. However, some studies have shown that activation of the Hh pathway inhibits osteoblastic differentiation of human mesenchymal stem cells (hMSCs)^10,12^. We hypothesize that post-transcriptional steps regulate the fine-tuning of the Hh pathway in adult stem cells. We are interested in understanding the post-transcriptional regulatory steps involved in the activation or blockage of the Hh pathway. In this study, we used well-characterized drugs, an activator (purmorphamine) and an inhibitor (cyclopamine) of the Hh pathway, which act by binding to the membrane protein SMO^13–15^, to treat adipose tissue-derived stem cells (ADSCs). We subsequently isolated and identified polysome-associated mRNAs from the ADSCs post treatment, and identified regulated genetic networks.

## Results

### GLI1 predominantly localized to the nucleus of ADSCs, regardless of Hh status

**Figure 1:**
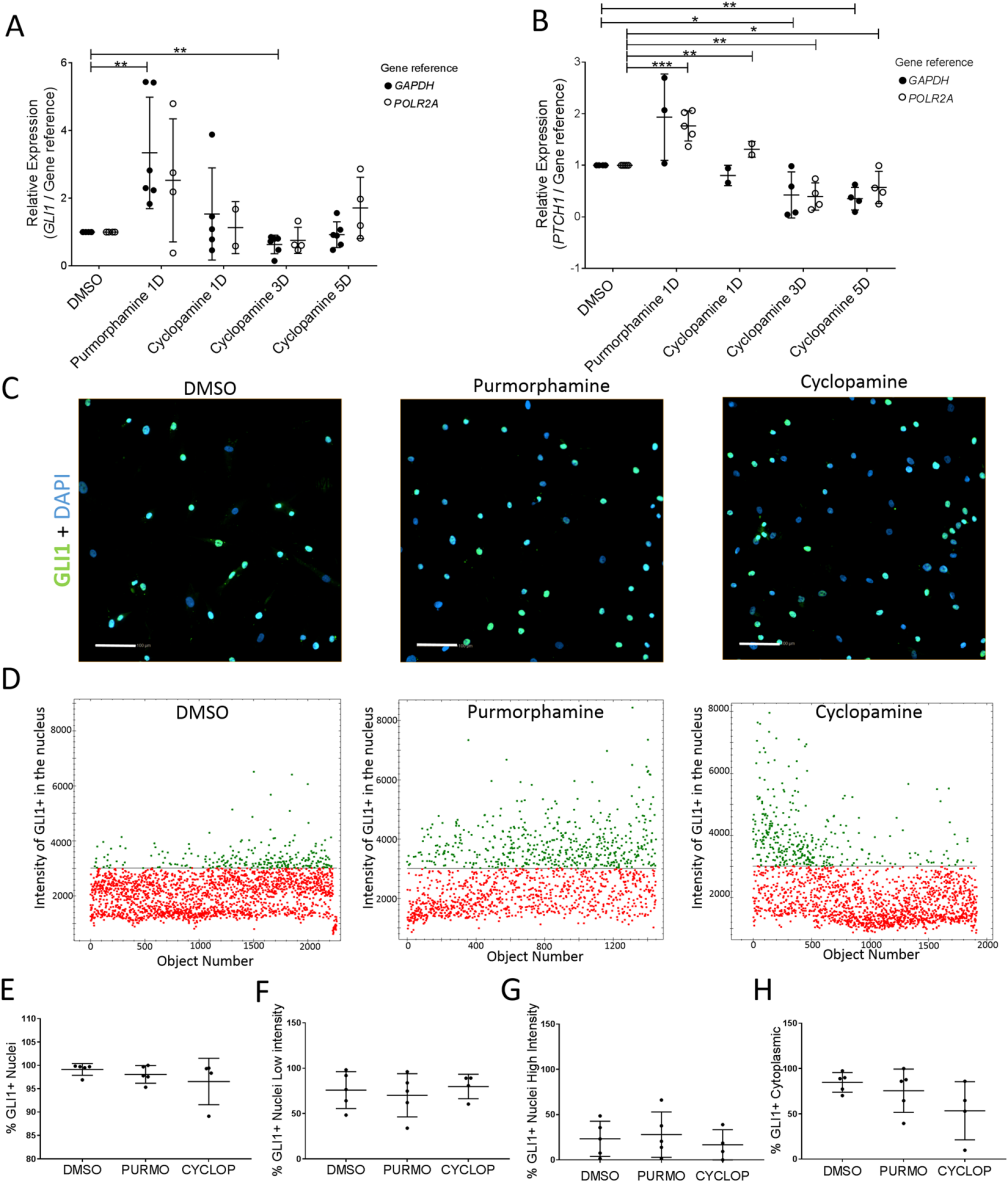
The transcriptional factor GLI1 is located in the nucleus of ADSCs. (**A**,**B**) qRT-PCR analysis of the level of GLI1 and PTCH1 mRNA in ADSCs treated with purmorphamine and cyclopamine during 1, 3 and 5 days; (**A**) *GLI1* mRNA (**B**) *PTCH1* mRNA. GAPDH and POLR2A were used as an internal housekeeping gene control. (Biological replicates = 2–6, each pont represent of the average of the technical triplicate, *P ≤ 0.05, **P ≤ 0.01, ***P ≤ 0.001). (**C**) Indirect immunofluorescence staining of GLI1 (green) in ADSCs after 24 h of DMSO, purmorphamine, or cyclopamine treatment. Nuclei were counterstained with DAPI (blue). Scale bar = 100 µm. (**D**) High-throughput imaging: GLI1+ staining intensity in the nucleus of ADSCs treated with DMSO, purmorphamine, and cyclopamine for 24 h. Object Number represents each cell that received a number according to the reading of the image. (**E**–**H**) Percentage of cells GLI1+ in to the nucleus and cytoplasm treated with DMSO (control), purmorphamine, and cyclopamine (n = 4–5). (**E**) Percentage of cells GLI1+ nuclei; (**F**) Percentage of cells GLI1+ Nuclei Low intensity; (**G**) Percentage of cells GLI1+ Nuclei High intensity; (**H**) Percentage of cells GLI1+ Cytoplasmic. There were no statistically significant differences between group means as determined by one-way ANOVA.

*GLI1* encodes a transcription factor that is activated and translocated to the nucleus in response to the Sonic Hh signal transduction cascade and regulates stem cell proliferation^16^. Here, we analyzed the association of mRNAs to polysomes at early steps (24 h) of Hh activation in ADSCs. First, we evaluated the conditions for activation or blocking of the Hh pathway in ADSCs by relative quantification of *GLI1* expression (Fig. 1A). After incubating the cells for one day with 1 µM of purmorphamine, we found that the level of *GLI1* expression increased nearly 3-fold and this effect was independent of drug concentration (Supplementary Figure 1A). Additionally, when cells were treated with 5 µM of cyclopamine^7^, the level of *GLI1* mRNA reduced after 3 days of treatment (Fig. 1A). Moreover, the expression level of *PTCH1*^17^, a GLI1 target gene, was evaluated by RT-qPCR and showed an expression profile very similar to GLI1 expression (Fig. 1B). This result suggests that cyclopamine acts late in downregulating *GLI1* expression in ADSC.

In order to evaluate whether the drugs affect the localization of GLI1, immunofluorescence was performed on ADSCs after 24 h of treatment with 5 µM cyclopamine and 1 µM purmorphamine (Fig. 1C,D). We found that ADSCs express the GLI1 protein in the nucleus without any exogenous ligand, this suggests that the Hedgehog signaling pathway is already activated in ADSCs. High throughput imaging analysis showed that although approximately 98% of the ADSC population express GLI1 protein in the nucleus, two well-defined and different populations were observed (Fig. 1C,D). One cell population showed a higher GLI1 immunostaining intensity (GLI1 + High) in the nucleus than the other (GLI1 + Low) (Fig. 1C,D). We also found that ADSCs treated with purmorphamine trend to show a higher percentage of cells with high-intensity GLI1 immunostaining in the nucleus compared with the controls (dimethyl sulfoxide, DMSO-treated) and cyclopamine-treated ADSCs, although there were no statistically significant differences (Fig. 1G,H, Supplementary Figure 1B–D). Furthermore, although cyclopamine did not cause changes in *GLI1* expression at the mRNA level after 24 h of treatment (Fig. 1A), there was a trend of a large number of cells with low GLI1 immunostaining intensity in the nucleus, although there were no statistically significant differences (Fig. 1F). The nuclear localization of GLI1 remained the same 3 and 5 days after purmorphamine and cyclopamine treatments (Supplementary Figure 1E). It seems a higher percentage of purmorphamine-treated cells were positive for GLI1 expression in the cytoplasm than cyclopamine-treated cells, although there were no statistically significant differences (Fig. 1H, Supplementary Figure 1D).

Hh and Nell-1 signaling exert additive effects on the proosteogenic and antiadipogenic differentiation of human adipose stem cells^7^. Thus, to determine if the location of GLI1 (the main transcriptional factor of the Hh pathway) is affected by differentiation; we induced the ADSCs to undergo adipogenesis and osteogenesis. Through immunofluorescence, we observed that GLI1 was predominantly localized in the nucleus in both differentiations (Supplementary Figure 1F).

### Differentially represented polysomal RNAs after 24 h of cell treatment with purmorphamine or cyclopamine

**Figure 2:**
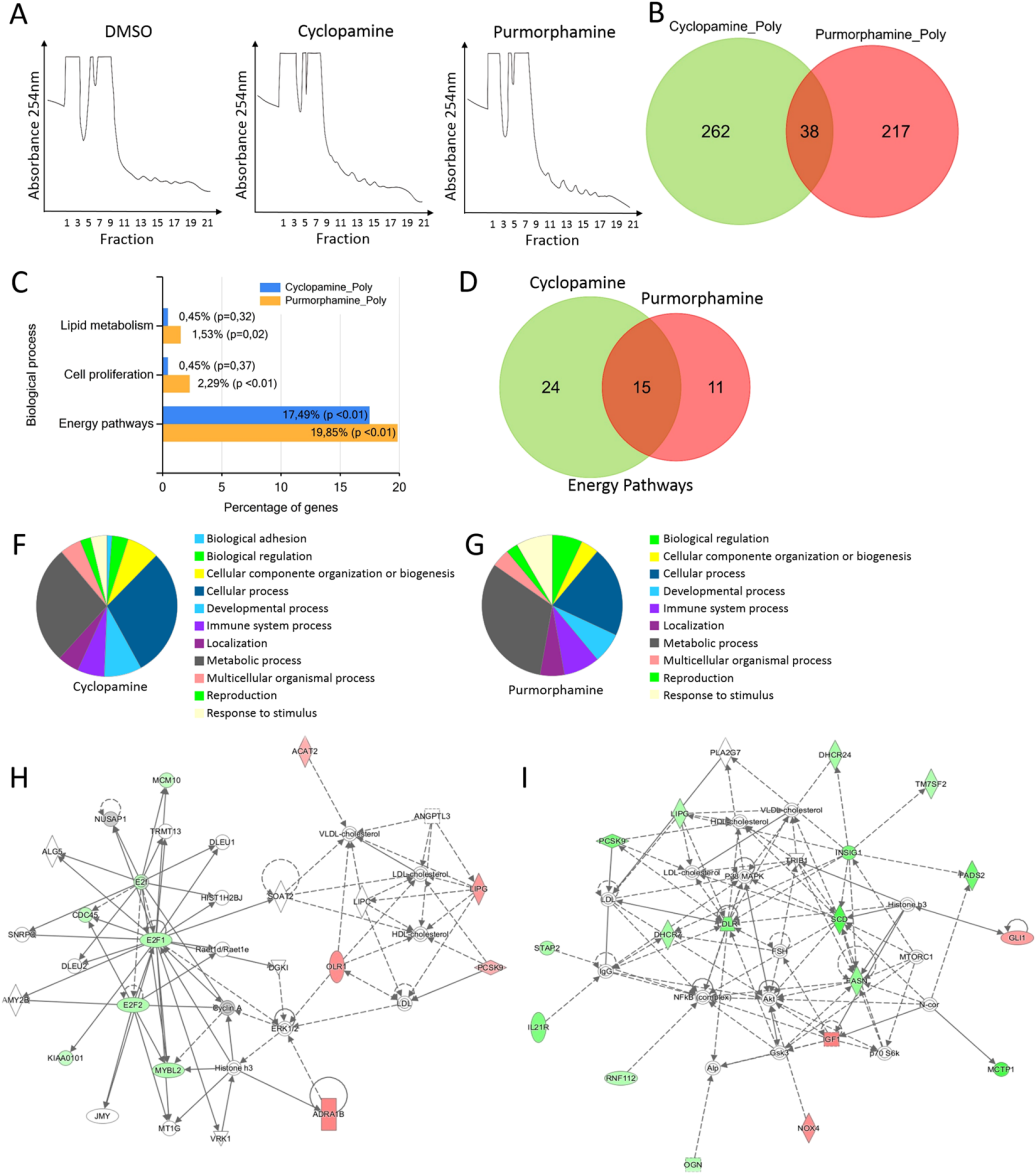
Different mRNAs associated with polysomes from ADSCs treated with cyclopamine and purmorphamine. (**A**) Polysomal profile of ADSCs treated with DMSO, cyclopamine, and purmorphamine. (**B**) Venn diagram shows the number of mRNAs associated with the polysomes in cells treated with cyclopamine and purmorphamine compared to that in cells treated with DMSO. (**C**) Gene ontology (Funrich) shows biological process of the set of mRNAs (Table S1), (**D**) Venn diagram shows the mRNAs in to Energy pathways term in each condition (**C**). **(F**,**G**) Gene ontology (Panther analysis) shows the biological processes involving the mRNAs associated with the polysomes in cells treated with (**F**) cyclopamine and (**G**) purmorphamine. (**H**,**I**) Representation of gene networks associated with lipid metabolism (Ingenuity Pathway Analysis) for both treatment: Cells treated with cyclopamine (**H**) and purmorphamine (**I**). Green: down-represented in polysomes. Red: up-represented in polysomes.

We then decided to investigate gene translation dynamics by polysome profiling in early time of Hh pathway modulation. The mRNAs associated with the translation machinery in ADSCs were identified using high-throughput sequencing of RNA-seq to determine the altered genetic networks by Hh pathway meddling. The polysome profile of ADSCs treated with both drugs showed no evident changes, compared with that of untreated cells (Fig. 2A). RNA from the polysome and ribosome-free fractions (mRNAs not associated with polysomes) was isolated and processed for large-scale sequencing (Supplementary Figure 2). RNA-seq samples were analyzed using a negative binomial distribution-based approach, with an FDR method for type II statistical error correction. An average of 95% of reads was successfully mapped to a human reference genome (hg38), corresponding to more than 50,000 detected transcripts per sample (genes and corresponding splicing isoforms). We identified 255 and 300 differentially represented mRNAs in the polysomal fraction (p-value ≤ 0.05), when cells were treated with purmorphamine and cyclopamine, respectively (Supplementary Table S1, Fig. 2B). These results demonstrated that although cyclopamine does not induce changes in *GLI1* expression at the mRNA level 24 h after treatment (Fig. 1A), the drug already affects the expression of the mRNAs in the polysomes (Fig. 2B). Purmorphamine-treated cells showed a higher amount of *GLI1* mRNA associated with polysomes compared with DMSO-treated cells, indicating that it is upregulated in the polysomes to be translated.

The functional enrichment of genes was carried out using FunRich tool^18^, and the analysis revealed that the differentially expressed genes under each condition were predominantly involved in energy pathways (Fig. 2C). Among the genes related to energy pathways, 15 genes were shared between the treatment groups (Lipase, endothelial *- LIPG*, Transmembrane 7 superfamily members 2 - *TM7SF2*, Squalene epoxidase *-SQLE*, 7 - dehydrocholesterol reductase *- DHCR7*, Mevalonate (diphospho) decarboxylase *- MVD*, Patatin-like phospholipase domain containing 3 - *PNPLA3*, Stearoyl-coa desaturase - *SCD*, Emopamil binding protein - *EBP*, 24-dehydrocholesterol reductase *- DHCR24*, Fatty acid desaturase 2 - *FADS2*, Fatty acid synthase - *FASN*, Star-related lipid transfer (START) domain containing 4 - *STARD4*, Atpase class V type 10D *- ATP10D*, Chromosome 14 open reading frame 1 - *C14orf1* and NADH dehydrogenase 1 alpha subcomplex, 3 *- NDUFA3*). However, 24 and 11 genes were unique to cyclopamine treatment group (Acetyl-coa acetyltransferase 2 - ACAT2, 3-hydroxy-3-methylglutaryl-coa synthase 1 - HMGCS1, 3-hydroxy-3-methylglutaryl-coa reductase -HMGCR, Cytochrome P450, family 51, subfamily A, polypeptide 1 - CYP51A1, Fatty acid desaturase 1 - *FADS1*, Hydroxysteroid (17-beta) dehydrogenase 8 - *HSD17B8*, Isopentenyl-diphosphate delta isomerase 1 - *IDI1*, Aldolase C, fructose-bisphosphate *- ALDOC*, Acyl-coa synthetase short-chain family member 2 - *ACSS2*, ATP citrate lyase *- ACLY*, Hydroxysteroid (17-beta) dehydrogenase 7 - *HSD17B7*, Farnesyl diphosphate synthase *- FDPS*, Farnesyl-diphosphate farnesyltransferase 1 - *FDFT1*, Mevalonate kinase *- MVK*, NAD(P) dependent steroid dehydrogenase-like *- NSDHL*, FK506 binding protein 4 *- FKBP4*, Retinol dehydrogenase 11 - *RDH11*, Pantothenate kinase 3 - *PANK3*, Hydroxysteroid (17-beta) dehydrogenase 12 - *HSD17B12*, Atpase family, AAA domain containing 2 - *ATAD2*, Thymidylate synthetase *- TYMS*, Prenyl (decaprenyl) diphosphate synthase, subunit 1 - *PDSS1*, Carbohydrate (N-acetylglucosamine 6-O) sulfotransferase 6 - *CHST6* and NADH dehydrogenase (ubiquinone) flavoprotein 2 -*NDUFV2*) and purmorphamine treatment group (NADPH oxidase 4 - *NOX4*, Cytochrome P450, family 26, subfamily B, polypeptide 1 - *CYP26B1*, Synapse differentiation inducing 1 - *SYNDIG1*, Transglutaminase 2 - *TGM2*, Dehydrogenase/reductase (SDR family) member 3 - *DHRS3*, Iduronate 2-sulfatase *- IDS*, Transketolase - *TKT*, Diazepam binding inhibitor - *DBI*, Phosphodiesterase 6 A *- PDE6A*, Carbonic anhydrase XI *- CA11* and Paraoxonase 3 - *PON3*), respectively (Fig. 2D). The Venn diagram representing the mRNAs involved in the energy pathways indicated that 38–57% of mRNAs associated to polysome are shared between treatments, and that 62% and 43% of the genes were exclusive to cyclopamine treatment and purmorphamine treatment, respectively (Fig. 2D). Gene ontology analysis using Panther^19^ shows that differently represented mRNAs associated with polysomes of cells when treated with both drugs has significant gene ontology terms indicating alterations in metabolic and cellular processes (Fig. 2F,G). We subsequently carried out Ingenuity Pathway Analysis (IPA) of the genes shown in Table S1, and it was observed that some genes from the generated genetic network were associated with lipid metabolism. They were inversely expressed in the treatments, confirming the robustness of the experiments (Fig. 2H,I). We also found within this genetic network two central elements corresponding to the E2F transcription factor 1 (*E2F1*) and 2 (*E2F2*). These results demonstrated that the amount of *E2F1* and *E2F2* mRNA associated with the polysomes was reduced in cyclopamine-treated cells.

**Figure 3:**
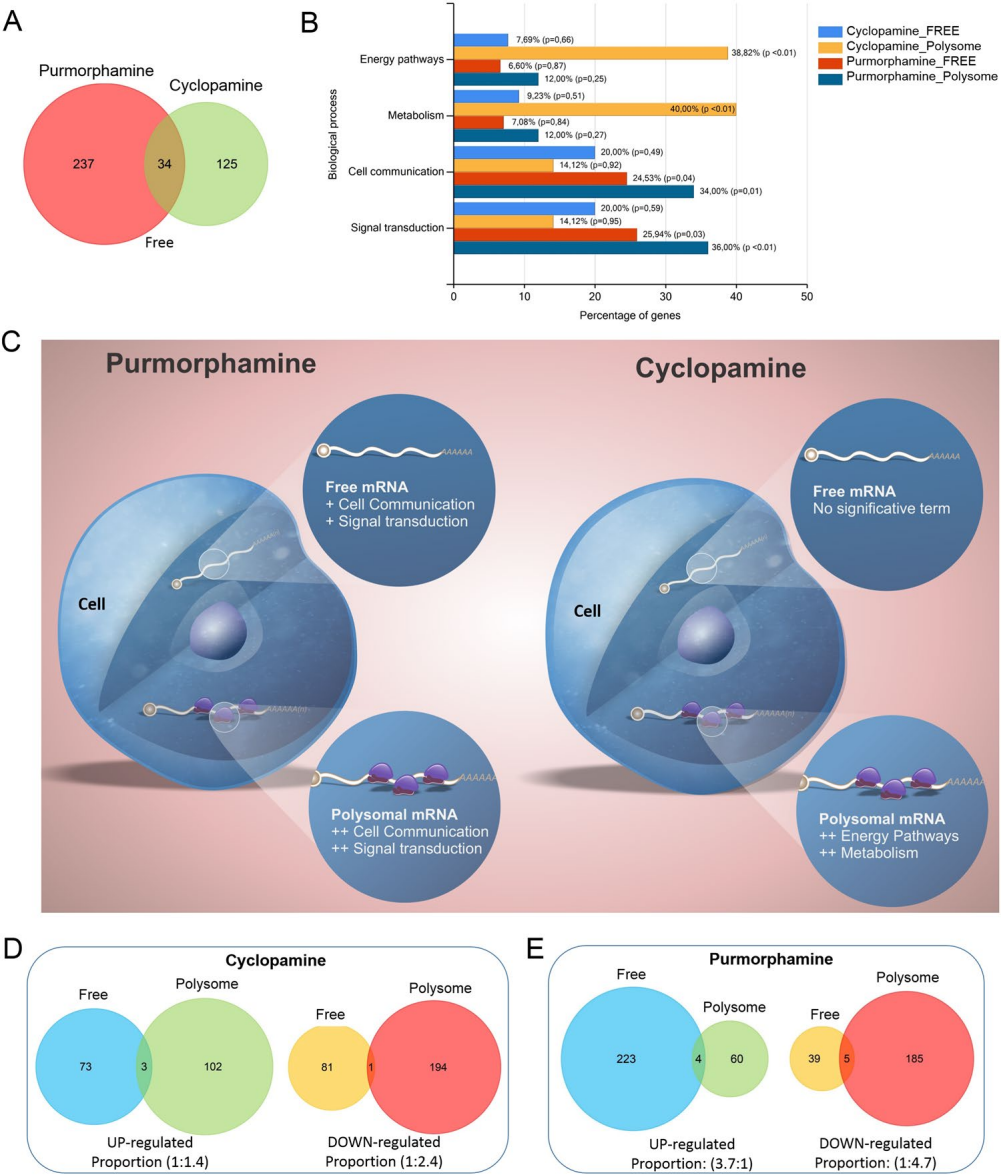
Comparison between differentially expressed mRNAs in free and polysomal fractions. (**A**) Venn diagram shows the number of differentially represented mRNAs in the ribosome-free fraction (mRNA not associated with polysome) from cells treated with cyclopamine and purmorphamine compared to that from cells treated with DMSO. (**B**) Comparative enrichment analyzes of the mRNAs that increased over the control (DMSO). (**C**) Scheme representing the free and polysomal fraction mRNA and their respective biological processes. (**D**,**E**) Venn diagram shows the number of up-represented and down-represented mRNAs in ADSCs treated with cyclopamine (**D**) and purmorphamine (**E**).

Next, we evaluated the dynamics of differentially represented mRNAs between ribosome-free fractions (mRNAs not associated with polysomes) and the polysome fractions of cells treated with purmorphamine and cyclopamine. The Venn diagrams (Fig. 3A) showed that treatment with cyclopamine resulted in a reduced level of mRNA expression in the free fraction compared to treatment with purmorphamine. In contrast, treatment with cyclopamine has more mRNAs associated with polysomes (Fig. 2B). These findings suggest the existence of different elements that could be regulating the dynamics of these mRNAs in the polysomes depending on the activation state of the Hh pathway.

Furthermore, a gene ontology analysis by Panther^19^ of mRNA groups that increased their representation in the polysomal and free fraction of cells, treated with cyclopamine and purmorphamine, was performed (Fig. 3B,C). We found that the cells treated with purmorphamine have mRNAs strongly involved in the cellular communication and signal transduction, although the ribosome-free mRNAs are also involved in these processes, but their expression level was low (Fig. 3B,C). When cells are treated with cyclopamine, polysome-associated mRNAs are related with energy pathways and metabolism.

When we clustered the groups of differentially represented mRNAs in the free and polysomal fractions for the different treatments (Fig. 3D,E), we observed that the proportion of mRNAs between the ribosome-free and polysomal fractions are similar in the upregulated and downregulated groups (Proportions 1: 1.4 and 1: 2.4, respectively) when treated with cyclopamine (Fig. 3D). Interestingly, this proportion of upregulated mRNAs in ribosome-free and polysomal fractions is inversely proportional to that of the mRNAs that were downregulated following purmorphamine treatment (Fig. 3E). In other words, purmorphamine treatment negatively regulates mRNA function by promoting the dissociation of mRNAs from the ribosomes, and increases their abundance in the ribosome-free fraction. This suggests that the activation of the Hh pathway by purmorphamine results in a negative regulation of the mRNAs in the protein translation machinery.

### *In silico* analysis suggests some candidate miRNAs for Hh pathway regulation

We hypothesized that there could be miRNAs that specifically regulated the group of polysome associated mRNAs induced by Hh. We grouped the mRNAs as follows: upregulated (UP-P) and downregulated (DOWN-P) mRNAs associated with the polysomes in cells treated with purmorphamine; and upregulated (UP-C) and downregulated (DOWN-C) genes in the polysomes in cells treated with cyclopamine. Genes common to theses tables and miRTarBase data (the experimentally validated microRNA-target interactions database^20^) are then shortlisted and stored in a new file containing information about the gene (i.e., miRNA, fold change, and PMID) (Table S2) in order to identify candidate miRNAs that target polysome-associated mRNAs (Supplementary Figure S3). Five miRNAs targeting mRNAs associated with the polysomes of cells treated with cyclopamine and purmorphamine are described in Table S3. In this table, we considered the number of identified targets for the miRNAs, and calculated the proportion of the total number of target mRNAs that respond to the Hh pathway. Thus, we observed some miRNAs, such as hsa-miR-3127-3p, hsa-miR6756-3p, hsa-miR150-5p, and hsa-miR215-5p, with proportions below 100, which regulated the Hh pathway to a greater extent. Moreover, we considered the number of mRNAs associated with the polysome, for example 67 upregulated mRNAs on cyclopamine treatment. Of these 67 mRNAs found to be associated with polysomes in cells treated with cyclopamine, 22 mRNAs are targets of hsa-miR335-5p. That is, 32.8% of that set of mRNAs that increases in the polysomes with the treatment with the drug cyclopamine were regulated by hsa-miR335-5p. Conversely, when we look at the DOWN-P group (161 mRNAs), 19 mRNAs were observed to be targets of hsa-miR335-5p. That is, 11.8% of this set of mRNAs decrease in the polysomes post the treatment that activates the Hh pathway. Thus, these *in silico* data show that hsa-miR335-5p may be a regulator during the activation or blockade of the Hh pathway.

## Discussion

Human MSCs are endowed with a basal Hh signaling activity that is necessary for efficient proliferation and clonogenicity of hMSCs^15^. Inhibition of Hh signaling with cyclopamine leads to a decrease in hMSC proliferation, arrest of the cells in the G0/G1 phases of the cell cycle^15^. Purmorphamine is a small molecule that is an agonist of Hh signaling^21^. Treatment of hMSCs with Sonic Hh or purmorphamine activates Hh signaling in turn inhibiting osteoblast differentiation^12^. Recent studies suggest that ADSCs are a heterogeneous mixture of cells containing subpopulations of stem and more committed progenitor cells^22^. The results of this study revealed two distinct populations presenting high and low nuclear GLI1 immunostaining intensity. This difference reflects the heterogeneity of the ADSC population *in vitro* or proliferation stage. This difference in basal GLI1 protein expression might perhaps explain the hererogeneity of ADSC responses to different inducers in the cell differentiation process. Virtually the entire ADSC population expresses GLI1 in the nucleus, and only a smaller portion expresses GLI1 in the cytoplasm. The nuclear import mechanism of GLI1 has been described for glioblastoma multiform cells. Forkhead box M1 (FOXM1) promotes nuclear import of GLI1 and thus increases the expression of its target genes^23^. The reason behind the cytoplasmic location of GLI1 in ADSCs treated with purmorphamine being smaller than the control may be due to this GLI1 import process by FOXM1. Interestingly, GLI1 mRNA expression in cyclopamine-treated ADSCs reduced only after the third day and with high concentrations of cyclopamine. Compared to other cell types such as Hela, for example, GLI1 mRNA expression reduced in 80% within 48 h of treatment with cyclopamine at a lower (16×) concentration^24^. The tolerance or reduced reactivity of ADSCs to some drugs could be due to FOXM1-induced drug resistance by regulating the transcription of *abcc5*, one of the ABC transporters, and controlling drug efflux^25^.

In previous work, we have shown that gene expression levels in the polysomal fraction reflect with more accuracy the population of mRNAs that are truly being translated into proteins. Changes at the total RNA levels in ADSCs are buffered by differential control of association of transcripts with polysomes. Moreover, most transcripts show regulation of their association with polysomes during cell differentiation [3-5]. In this work, we decided to focus on the polysomal associated population of mRNAs as we have shown that this level of gene expression control is essential for cell differentiation. We found *E2F1* and *E2F2* to be downregulated regulatory elements of the network involved with lipid metabolism in cyclopamine-treated ADSCs, and their downregulation occurred before the reduction of GLI1 mRNA expression (Figs 1B and 2H). E2F1 is a crucial mediator of Hh signaling, and it is required for melanoma cell proliferation and xenograft growth induced by activation of the Hh pathway^26,27^. The modulation of the Hh pathway affects the expression of *E2F1* and *E2F2* mRNAs and also their association with the polysomes and this appears to be and early regulatory event before the down regulation of *GLI1* mRNA.

**Figure 4:**
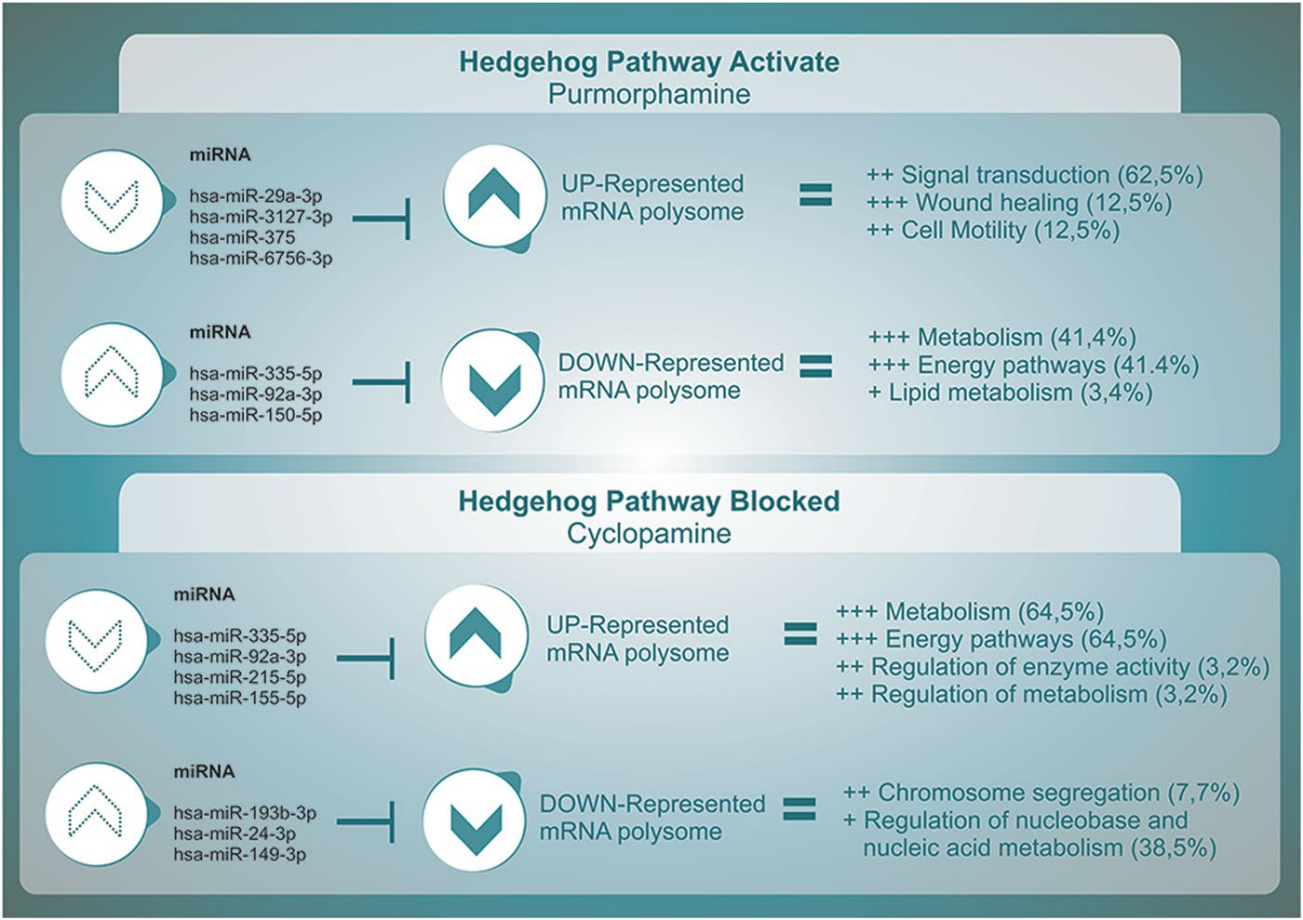
Scheme representation of the relation between candidate miRNAs and differentially represented target mRNAs associated with the polysomes, and the biological processes in which these mRNAs are involved (Funrich).

Once mRNAs are transcribed, spliced and transported to the cytoplasm, their fate is determined by the complex interplay of RNA binding proteins and microRNAs (miRNAs) that act on regulatory elements within the transcripts^28^. The observed mRNAs at the polysomal level allowed to determine possible candidate miRNAs participating in the Hh pathway (Fig. 4). Several studies demonstrated the relation of the activation of the Hh pathway to the development or progression of different types of cancer^26,29–32^. Among the miRNA identified in this study we found several related to regulation of cell proliferation and tumoral development. Overexpression of miR-335 induces cell proliferation and tumor growth to colorectal carcinoma cells^33^. Although, miR-335 has been identified as a metastasis-suppressing microRNA in human breast cancer^34^, it also suppresses the proliferation, and invasion of breast cancer cells by targeting EphA4^35^. These variations may be due to different types of cancer, their stages, or even molecular scenario intrinsic to each type of cell. The presence of miR-92a in serum or plasma is associated with acute myeloid leukemia, colorectal cancer, and small lung cancer^36–38^. miR-92a promotes tumor growth of osteosarcoma^39,40^ and proliferation and invasion in hepatocellular carcinoma cells^41^. miR-215 promotes cell migration and invasion of gastric cancer cells, and acts as an oncogene in high-grade glioma by regulating retinoblastoma 1^42,43^, even though, it suppresses proliferation and migration of non-small cell lung cancer cells and epithelial ovarian cancer cells^44,45^. miR-150 is downregulated in osteosarcoma and suppresses cell proliferation, migration, and invasion^46^. miR-150 can induce cell cycle arrest at G0/G1 phase and weaken proliferation of esophageal carcinoma cells via targeted inhibition on GLI1^47^, even though, it promotes cellular metastasis in non-small cell lung cancer by targeting Forkhead box O4 (FOXO4)^48^. Our data shows that hsa-miR-29a, hsa-miR-3127, hsa-miR-375, hsa-miR-6756, hsa-miR-335, hsa-miR-92a, hsa-miR-150, hsa-miR-215, hsa-miR-155, hsa-miR-193b, hsa-miR-24, and hsa-miR-149 are strong candidates for further studies on the Hh signaling pathway in ADSCs.

We showed that GLI1 is expressed in the nucleus of virtually the entire population of ADSCs. Purmorphamine activates the Hh pathway in ADSCs, and after only 24 h of treatment, it affects the expression of the GLI1 mRNA. Cyclopamine blocks the Hh pathway in ADSCs, but the response is slower, affecting GLI1 expression at the mRNA level after three days. The effect of purmorphamine and cyclopamine on ADSCs was monitored at the post-transcriptional level, where we identified mRNAs associated with polysomes. Activation of the Hh pathway by purmorphamine results in increased mRNAs associated with cellular communication and signal transduction. Blockade of the Hh pathway by cyclopamine leads to increased mRNAs associated with metabolism and energy pathways. Our results suggest that activation of the Hh pathway by purmorphamine also results in a negative regulation of the mRNAs in the protein translation machinery. In silico, we identified potential miRNAs, such as miR335 that regulated these mRNAs associated with polysomes and modulated the Hh pathway.

## Methods

### Cell culture

Human ADSCs from lipoaspirates were purchased from a commercial source (Lonza, Walkersville, USA; catalog number PT-5006). ADSCs were cultured in accordance with the datasheet instructions. In brief, cells were cultured in Dulbecco’s modified Eagle medium (DMEM) (Gibco Invitrogen, Carlsbad, CA, USA) supplemented with 10% fetal bovine serum (Gibco Invitrogen, Carlsbad, CA, USA) and 4 mM L-glutamine (Gibco Invitrogen, Carlsbad, CA, USA), without antibiotics. The cells were maintained at 37 °C in 5% CO_2_ atmosphere. Cyclopamine (Sigma Aldrich - St. Louis, MO, EUA) was dissolved in dimethyl sulfoxide (DMSO), having a stock concentration of 1 mM and used at a final concentration of 5 µM in culture for 24 h. Purmorphamine (Sigma Aldrich - St. Louis, MO, EUA) was dissolved in DMSO, having a stock concentration of 1 mM and at a final concentration of 1 µM in culture for 24 h.

### Quantitative reverse transcription-polymerase chain reaction (RT-qPCR)

RT-qPCR was performed using the 7500 Fast Real Time PCR Systems v2.0.6 (Applied Biosystems - Foster City, CA, EUA). Amplifications were carried out in a final reaction volume of 20 µL using the SYBR Green master mix (Applied Biosystems - Foster City, CA, EUA), 100 ng cDNA template, and 5–10 pmol primers. PCR conditions were 95 °C for 10 mins, followed by 45 cycles of 95 °C for 30 s, gene specific annealing temperature for 30 s, and 72 °C for 40 secs. The melting curves were acquired after PCR to confirm the specificity of the amplified products. The primers used for Glyceraldehyde-3-phosphate dehydrogenase - GAPDH F 5′GGCGATGCTGGCGCTGAGTAC3′ and R 5′TGGTTCACACCCATGACGA3′, annealing temperature of 60 °C and 149 base pairs; GLI1 F 5′CCCGCCCTTCTGCCACCAAG3′ and R 5′ACCGTCTGCAGGTCCAGGCT3′, annealing temperature of 62 °C and 182 base pairs; Polymerase (RNA) II (DNA directed) polypeptide A - POLR2A F 5′TACCACGTCATCTCCTTTGATGGCT3′ and R 5′GTGCGGCTGCTTCCATAA3′, annealing temperature of 60 °C and 186 base pairs, PTCH1 F 5′ATCCATGTGGCTGCCCTCTT3′ and R 5′CACAGCTCCTCCACGTTGGT3′, annealing temperature of 60 °C and 223 base pairs. We generated standard curves for each gene, including the control (housekeeping) gene. The GAPDH and POLR2A transcripts were used as an internal control. Amplifications were performed with technical triplicates and 2-5 biological replicates. Student’s *t*-test was used to assess the significance of differences between the cell populations. We considered P values < 0.05 statistically significant.

### Indirect Immunofluorescence

Cells seeded on glass coverslips were fixed in 4% formaldehyde solution for 10 min and washed with PBS. Subsequently, they were permeabilized using 0.5% Triton X-100 in PBS for 10 min. Nonspecific binding sites were blocked with 3% BSA for 1 h, and the cells were then incubated for 3 h at 37 °C with anti-GLI1 antibody diluted (1:50) in PBS containing 1% BSA, and subsequently washed with PBS. The cells were incubated for 1 h at 37 °C with secondary antibody Anti-Rat IgG–FITC produced in goat at a dilution of 1:20. Cell nuclei were stained with DAPI. Images were obtained using a Nikon E- 600 microscope. The cells were counted automatically from 25 selected fields per coverslips.

### Quantification of GLI1 expression by High Content Imaging System

The GLI1 quantification assay was acquired using a High Content Imaging System (Operetta CLS, Perkin Elmer). A total of 25 photos were acquired per field per coverslips at a magnification of 20x, 4-5 biological replicates. For nuclei quantification, the images were acquired on DAPI Channel (excitation of 355–385 nm and emission of 430–500 nm) with 5 milliseconds of exposure. Were considered in this analysis nuclei with a roundness > 0.9 in order to exclude cell debris. For the analysis of GLI1+ cells, the images were acquired on Alexa 488 Channel (excitation of 460–490 nm and emission of 500–550 nm) with 200 milliseconds of exposure, 50% power for both fluorochromes. Quantification of nuclear immunostaining, enumeration of GLI1+ cells, and determination of GLI1 immunostaining intensity were performed using Operetta CLS and Analysis Software 4.5 (Perkin Elmer). Data were expressed as number of nuclei per well, % GLI1+ cells, and low or high intensity GLI1 immunostaining. Based on image analysis, an intensity above 1000 was defined as the cut-off line between negative and positive staining for GLI1; an intensity between 1001 and 3000 was considered low and values above 3001 were considered high.

### Polysome profiling and RNA purification

Polysomal fractions from ADSC cultures treated with cyclopamine, purmorphamine, or DMSO for 24 h were prepared using the method described previously^49^. Total number of cells per assay was of 4.6 × 10^6^ (±1.3) which render 24.4 µg (±1.6) of cytoplasmic RNA. In brief, cells were treated with 0.1 mg/mL cycloheximide (Sigma-Aldrich) for 10 min at 37 °C, removed from the culture flasks with a cell scraper, and resuspended in 0.1 mg/mL cycloheximide in PBS. The suspension was centrifuged (2,000 × g for 5 min), and the resulting pellet was washed twice with 0.1 mg/mL cycloheximide in PBS. The cells were lysed by incubation for 10 min on ice with polysome buffer (15 mM Tris-HCl pH 7.4, 1% Triton X-100, 15 mM MgCl_2_, 0.3 M NaCl, 0.1 mg/mL cycloheximide), and the cell lysate was centrifuged at 12,000 × g for 10 min at 4 °C. The supernatant was carefully isolated, loaded onto 10–50% sucrose gradients, and centrifuged at 39,000 rpm (HIMAC CP80WX HITACHI) for 160 min at 4 °C. The sucrose gradient was fractionated with the ISCO gradient fractionation system (ISCO Model 160 gradient former), connected to a UV detector to monitor the absorbance at 275 nm, and the polysome profile was recorded. The free and polysomal RNA fractions were extracted by DirectZOL (Zymo Research).

### Large-scale sequencing

RNA from free and polysomal fractions were extracted using DirectZol kit (Zymo). The sample will be prepared for sequencing in Illumina Platform using TruSeq Stranded Total RNA LT Kit. For clustering and sequencing used TruSeq SR Cluster Kit v3 - cBot – HS and TruSeq SBS Kit v3 - HS (100-cycles). This process generates per reaction, on average, 30 million sequenced fragments of size between 85–100 nucleotides (called reads). The raw data were deposited in the ArrayExpress under the number: E-MTAB-6254.

### Differential gene expression analysis

Sequenced translatome data was filtered for high quality read retrieval, by removing low quality score data and potential artifacts, and sequencing contaminants using Cutadapt software^50^. Next, all replicates of RNA-seq data were mapped to human reference genome using STAR aligner^51^, and using a read-count methodology, the absolute number of aligned reads for each assembled transcript were separately extracted from each alignment files and represented in a count matriz by the software HTSeq-count^52^. The rows represent transcripts and columns represent fragment counts for a specific sample. To reach statistical significance, the read counting matrix was subjected to a negative binomial distribution approach by applying an statistical adjusting method proposed by Benjamini & Hochberg^53^, the False Discovery Rate (FDR) correction. This approach is used to control false-positive significance in transcript expression variation. The statistical analysis were conducted in our bioinformatics pipeline by using the R Bioconductor package DESeq. 2^54^. Differential expression between different samples was detected for those genes with statistical significance, P value < 0.05. Differentially expressed genes were also analyzed using Blast2GO for GO annotation^55^. Quantitative differences in differentially expressed transcripts were represented by Venn diagrams to show common or exclusive genes among samples. The count matriz for all sequenced samples was also used to calculate and generate a Euclidian distance matrix for hierarchical sample clustering to group samples according to the transcriptome profile. We also performed an additional analysis using the established analysis pipelines Xtail^56^ for RNA-seq data generated from polysome profiles. Alignment data from polysomal data was restricted to protein-coding open reading frame (ORF), and repetitive alignments were ignored according to the reccomended Xtail protocol. Significant expression alterations for polysomal analysis were restricted for p-value < 0.05, with Falso discovery rate (FDR) statistical analysis also included (Supplementary Table S4).

### Statistical Analysis

Statistical analyses were performed on data obtained from at least three independent experiments carried out with ADSCs from three different donors in biological replicates. Results are presented as mean ± SD. The significance of the differences observed was evaluated by Student’s *t*-test. P values < 0.05 were considered as statistically significant.

### Ethical Approval and Consent to participate

This study is accordance with the guidelines for research involving human subjects, and with the approval of the Ethics Committee of *Fundação Oswaldo Cruz*, Brazil (CAAE: 48374715.8.0000.5248).

## Electronic supplementary material

SUPPLEMENTARY FIGURES and Table S3

Table Supplementary S1

Table Supplementary S2

Table Supplementary S4
