# Supplementary material for: Crosstalk between Hedgehog pathway and energy pathways in human adipose-derived stem cells: A deep sequencing analysis of polysome-associated RNA
Source: Sci Rep. 2018 May 30;8:8411. doi: 10.1038/s41598-018-26533-y (PMC5976649; doi:10.1038/s41598-018-26533-y)
Supplement: Supplementary file 1 — SUPPLEMENTARY FIGURES and Table S3 [file 41598_2018_26533_MOESM1_ESM.docx]

**SUPPLEMENTARY FIGURES**

**Crosstalk between Hedgehog pathway and energy pathways in human adipose-derived stem cells: A deep sequencing analysis of polysome-associated RNA**

Patrícia Shigunov^1*^, Lucas Titton Balvedi^1^, Marlon Dias Mariano Santos^2^, Roberto H. Herai^3^, Alessandra Melo de Aguiar^1^, Bruno Dallagiovanna^1^

**
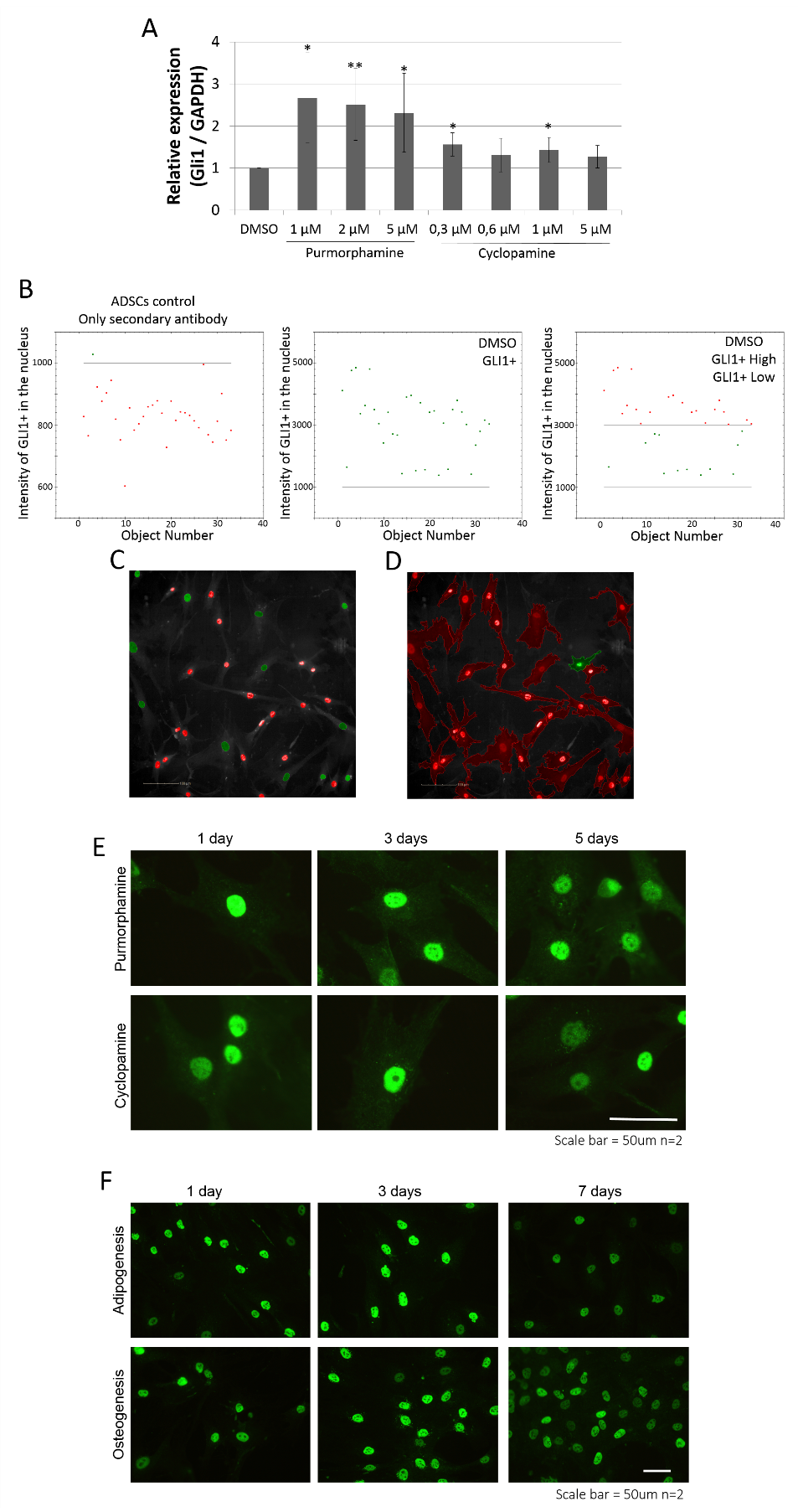
**

**Figure Supplementary S1**. (A) qRT-PCR analysis of the level of GLI1 mRNA in ADSCs treated with different concentrations of purmorphamine and cyclopamine (B) Analysis of the images to determine the GLI1 immunostaining intensity in the nucleus of ADSCs. (C) Representative image of the filters applied by the software for population with high- (green) and low- (red) intensity GLI1 expression. (D) Representative image of the filters applied by the software to determine the cells with GLI1 expression in the cytoplasm; Cytoplasmic GLI1+ (Green) and GLI1- (Red). (E) GLI1 localization in ADSCs treated with purmorphamine and cyclopamine for 1, 3, and 5 days (n = 2). (F) GLI1 localization in ADSCs induced adipogenesis and osteogenesis for 1, 3, and 7 days (n = 2).

**
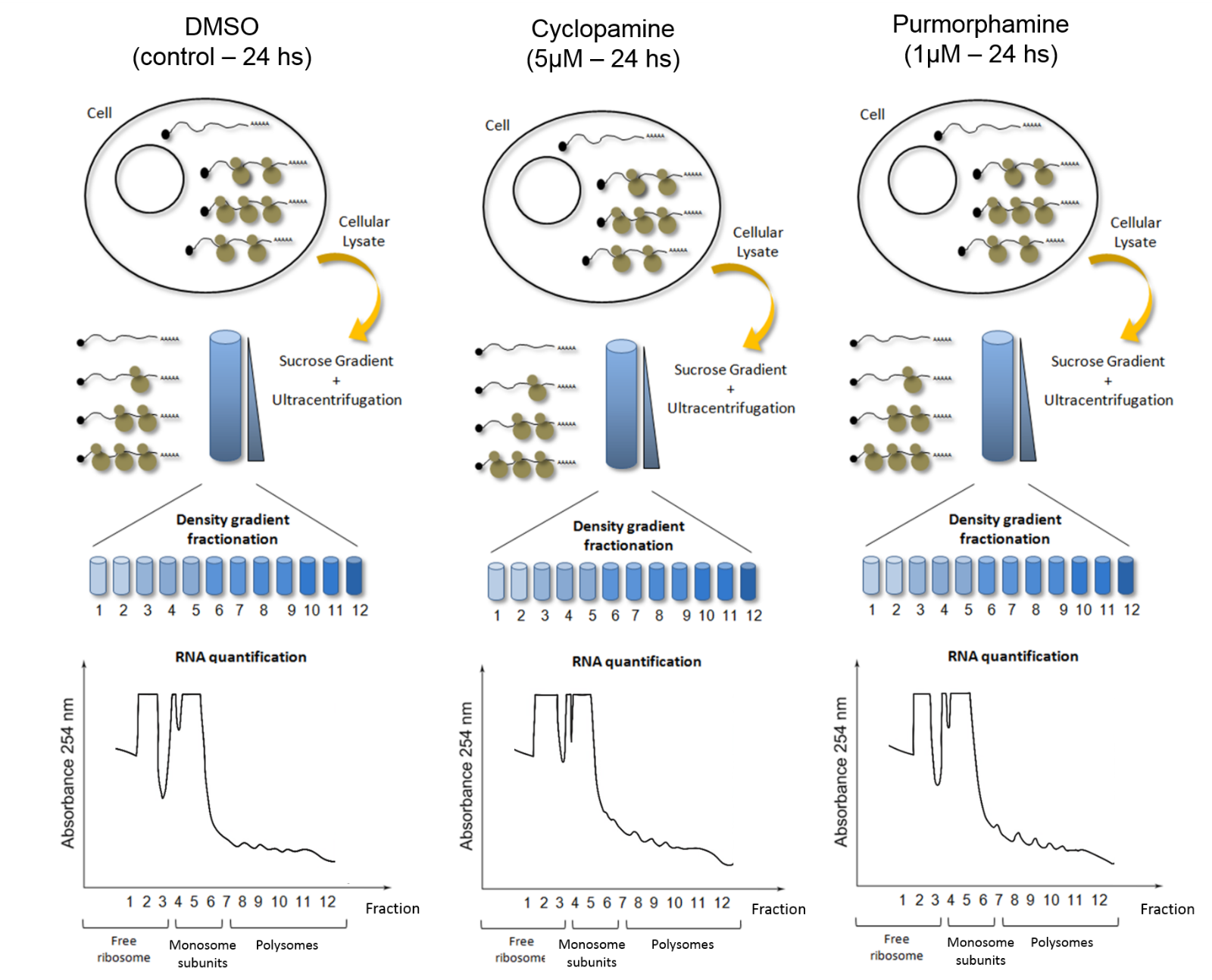
Figure Supplementary S2**. Schematic representation of the isolation of free and polysomal RNAs (n = 3).

**
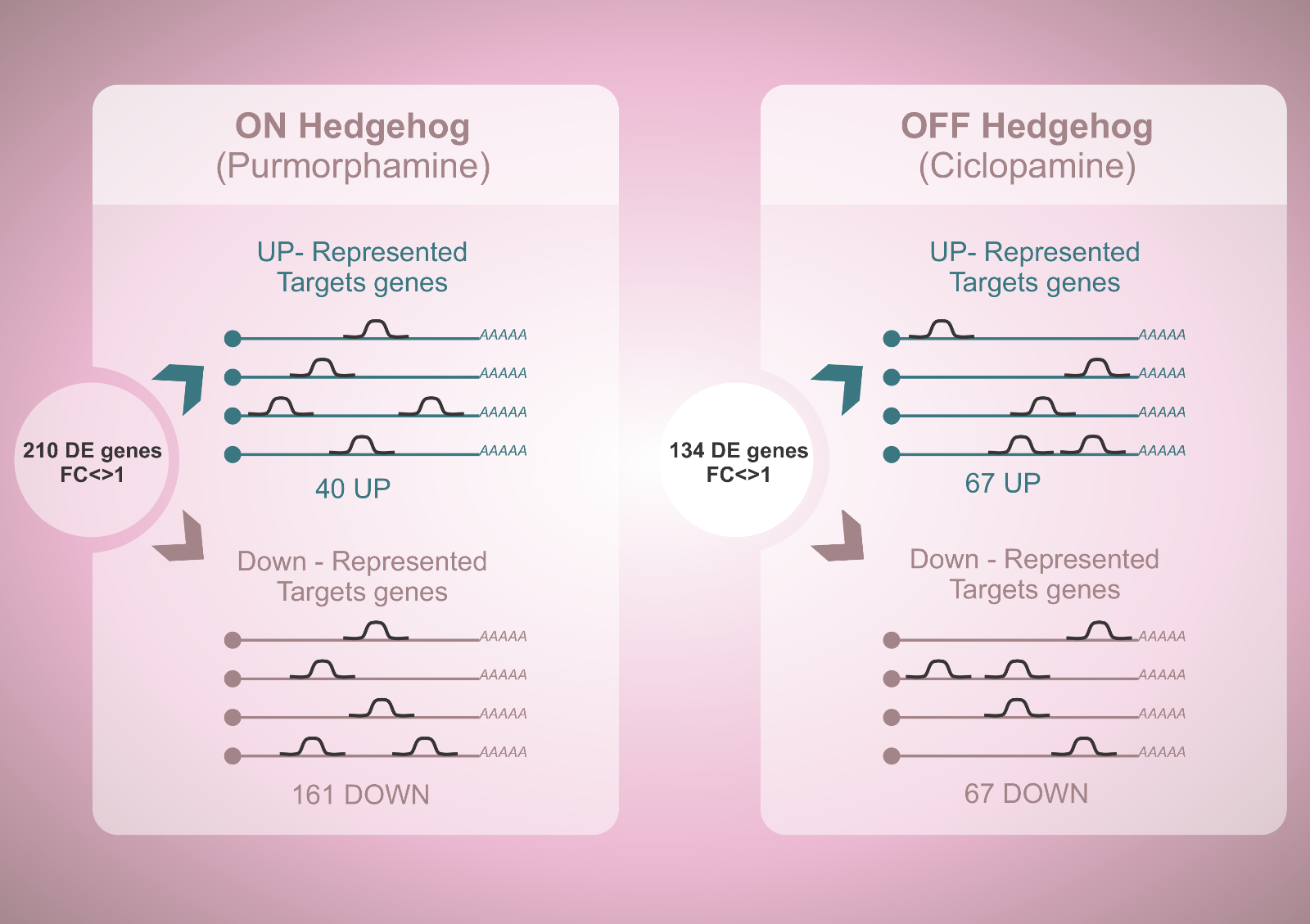
Figure Supplementary S3.** Scheme of the strategy to identify possible miRNAs candidates that target mRNAs regulated in the polysomes with the treatments with cyclopamine and purmorphamine. Genes without “gene name” were not considered, then the sum of the up-represented and down-represented genes does not close the total of differentially represented mRNAs.

**Table Supplementary S1** – Excel spreadsheets

**Table Supplementary S2** – Excel spreadsheets

**Table Supplementary S3**

Table S3. Representation of the five most-targeted miRNAs in the polysomes of cells treated with cyclopamine and purmorphamine.

| **CYCLOPAMINE** | | | | | | |
| --- | --- | --- | --- | --- | --- | --- |
| **Group** | **miRNA (hsa)** | **Total targets (miRTarbase)** | **Number of Polysome targets** | **Proportion (Polysome targets : Total targets)** | **Proportion (Polysome targets: Total Group)** | **Polysome targets (Gene symbol)** |
| UP – C  67 mRNAs | **miR-335-5p #** | 2647 | 22 | 1:120 | 1:3 | SLCO4C1; LIPG; TM7SF2; PCSK9; INSIG1; DHCR24; HMGCS1; SQLE; DHCR7; GDPD1; HMGCR; PNPLA3; CYP51A1; FADS1; SCD; MSMO1; IL21R; ALDOC; ACSS2; FADS2; HSD17B7; LDLR; |
|  | **miR-192-5p** | 1024 | 10 | 1:102 | 1:7 | MT1F; HES6; IDI1; PCSK9; INSIG1; DHCR24; HMGCS1; FADS1; SCD; PODXL; |
|  | **miR-92a-3p #** | 1639 | 10 | 1:164 | 1:7 | LDLR; ACLY; ACAT2; EBP; INSIG1; DHCR24; HMGCS1; HMGCR; SCD; FASN; |
|  | **miR-215-5p** | 767 | 9 | 1:85 | 1:7 | MT1F; HES6; PODXL; PCSK9; INSIG1; DHCR24; IDI1; FADS1; SCD; |
|  | **miR-155-5p** | 1157 | 8 | 1:145 | 1:8 | CYP51A1; PODXL; HSD17B7; OLR1; DHCR24; HMGCS1; FADS1; SCD; |
| DOWN – C  67 mRNAs | **miR-193b-3p** | 927 | 8 | 1:116 | 1:8 | C1QTNF2; KIAA0101; MCM10; WDR62; E2F1; E2F2; SPC25; MND1; |
|  | **miR-24-3p** | 892 | 6 | 1:149 | 1:11 | HIST3H2BB; KIAA0101; MCM10; PRRG4; E2F1; E2F2; |
|  | **miR-149-3p** | 738 | 5 | 1:148 | 1:13 | ESCO2; CLSPN; MYBL2; PRRG4; E2F1; |
|  | **miR-192-5p** | 1024 | 5 | 1:205 | 1:13 | FAM111B; CLSPN; MCM10; PRRG4; ESCO2; |
|  | **miR-26b-5p #** | 1964 | 5 | 1:393 | 1:13 | CDCA3; KIAA0101; SPC25; PLCB2; FBXO24; |
| **PURMORPHAMINE** | | | | | | |
|  | **miRNA (hsa)** | **Total targets (miRTarbase)** | **Number of Polysome targets** | **Proportion (Polysome targets : Total targets)** | **Proportion (Polysome targets: Total Group)** | **Polysome targets (Gene symbol)** |
| UP – P  40 mRNAs | **miR-124-3p *** | 1704 | 3 | 1:568 | 1:13 | F3; FSTL3; SGK1; |
|  | **miR-29a-3p *** | 313 | 3 | 1:104 | 1:13 | NEDD9; IGF1; SGK1; |
|  | **miR-3127-3p** | 173 | 3 | 1:58 | 1:13 | NEDD9; APLN; PMEPA1; |
|  | **miR-375** | 495 | 3 | 1:165 | 1:13 | F3; FSTL3; CCDC88B; |
|  | **miR-6756-3p** | 190 | 3 | 1:63 | 1:13 | NEDD9; APLN; PMEPA1; |
| DOWN –P  161 mRNAs | **miR-335-5p #** | 2647 | 19 | 1:139 | 1:8 | LDLR; PLEKHB1; DHCR24; SREBF2; SCD; INSIG1; PCSK9; DHCR7; SQLE; PNPLA3; CACNA2D4; IL21R; LIPG; CDR1; FADS2; TM7SF2; NRIP2; PON3; ACE; |
|  | **miR-92a-3p #** | 1639 | 10 | 1:164 | 1:16 | LDLR; PLEKHB1; DHCR24; SREBF2; SCD; INSIG1; FASN; ZNF551; TTC32; SCARB1; |
|  | **miR-124-3p *** | 1704 | 7 | 1:243 | 1:23 | LDLR; PLEKHB1; DHCR24; PYCARD; INO80C; MVD; PCSK9; |
|  | **miR-150-5p** | 548 | 6 | 1:91 | 1:27 | PDE6A; LIPG; PNPLA3; ZNF551; FAM213A; PLXDC1; |
|  | **miR-26b-5p #** | 1964 | 6 | 1:327 | 1:27 | PNPLA3; MVD; CA11; ZNF551; FADS2; PACRG; |
|  | # miRNA than regulated genes in both treatments (cyclopamine and purmorphamine). | | | | | |
|  | * miRNA described in the literature with function in the hedgehog pathway.  **Abbreviations**  has – Homo sapiens  UP-C – Transcripts Up-represented in polysome of cells treated with cyclopamine.  DOWN-C – Transcripts Down-represented in polysome of cells treated with cyclopamine.  UP-P – Transcripts Up-represented in polysome of cells treated with purmorphamine.  DOWN-P – Transcripts Down-represented in polysome of cells treated with purmorphamine. | | | | | |

**Table Supplementary S4 -** Excel spreadsheets
